# Correlation of microscopic tumor extension with tumor microenvironment in esophageal cancer patients

**DOI:** 10.1007/s00066-024-02234-6

**Published:** 2024-05-10

**Authors:** Benjamin Terfa Igbo, Christina Jentsch, Annett Linge, Ioana Plesca, Yalçin Kuzay, Steffen Löck, Mani Sankari Kumaravadivel, Susanne Doms, Liane Stolz-Kieslich, Daniela Pollack, Sascha Brückmann, Hannes Tittlbach, Jürgen Weitz, Daniela Aust, Rudi Apolle, Marc Schmitz, Esther G. C. Troost

**Affiliations:** 1 Institute of Radiooncology—OncoRay, Helmholtz-Zentrum Dresden-Rossendorf, Dresden, Germany; 2 OncoRay—National Center for Radiation Research in Oncology, Faculty of Medicine and University Hospital Carl Gustav Carus, Technische Universität Dresden, Helmholtz-Zentrum Dresden-Rossendorf, Dresden, Germany; 3 Department of Radiotherapy and Radiation Oncology, Faculty of Medicine and University Hospital Carl Gustav Carus, Technische Universität Dresden, Fetscherstraße 74, 01307 Dresden, Germany; 4 German Cancer Research Center (DKFZ) and German Cancer Consortium (DKTK), Partner Site Dresden, Dresden, Germany; 5 National Center for Tumor Diseases (NCT/UCC), Dresden, Germany, German Cancer Research Center (DKFZ), Heidelberg; Faculty of Medicine and University Hospital Carl Gustav Carus, Technische Universität Dresden, Dresden, Germany; Helmholtz-Zentrum Dresden-Rossendorf (HZDR), Dresden, Germany; 6 Institute of immunology, Faculty of Medicine Carl Gustav Carus, Technische Universität Dresden, Dresden, Germany; 7 Department of Visceral, Thoracic and Vascular Surgery (VTG), Faculty of Medicine and University Hospital Carl Gustav Carus Dresden, Dresden, Germany; 8 Institute for Pathology, Faculty of Medicine and University Hospital Carl Gustav Carus, Technische Universität Dresden, Dresden, Germany; 9 Institute for Pathology and Tumor and Normal Tissue Bank of the University Cancer Center (UCC), Faculty of Medicine and University Hospital Carl Gustav Carus, Technische Universität Dresden, Dresden, Germany

**Keywords:** Immunohistochemical analysis, Multiparametric analysis, Hypoxia, Proliferation, Tumor stem cells, Clinical Target Volume

## Abstract

**Objective:**

In the era of image-guided adaptive radiotherapy, definition of the clinical target volume (CTV) is a challenge in various solid tumors, including esophageal cancer (EC). Many tumor microenvironmental factors, e.g., tumor cell proliferation or cancer stem cells, are hypothesized to be involved in microscopic tumor extension (MTE). Therefore, this study assessed the expression of FAK, ILK, CD44, HIF-1α, and Ki67 in EC patients after neoadjuvant radiochemotherapy followed by tumor resection (NRCHT+R) and correlated these markers with the MTE.

**Methods:**

Formalin-fixed paraffin-embedded tumor resection specimens of ten EC patients were analyzed using multiplex immunofluorescence staining. Since gold fiducial markers had been endoscopically implanted at the proximal and distal tumor borders prior to NRCHT+R, correlation of the markers with the MTE was feasible.

**Results:**

In tumor resection specimens of EC patients, the overall percentages of FAK^+^, CD44^+^, HIF-1α^+^, and Ki67^+^ cells were higher in tumor nests than in the tumor stroma, with the outcome for Ki67^+^ cells reaching statistical significance (*p* < 0.001). Conversely, expression of ILK^+^ cells was higher in tumor stroma, albeit not statistically significantly. In three patients, MTE beyond the fiducial markers was found, reaching up to 31 mm.

**Conclusion:**

Our findings indicate that the overall expression of FAK, HIF-1α, Ki67, and CD44 was higher in tumor nests, whereas that of ILK was higher in tumor stroma. Differences in the TME between patients with residual tumor cells in the original CTV compared to those without were not found. Thus, there is insufficient evidence that the TME influences the required CTV margin on an individual patient basis.

**Trial registration number and date:**

BO-EK-148042017 and BO-EK-177042022 on 20.06.2022, DRKS00011886, https://drks.de/search/de/trial/DRKS00011886.

**Supplementary Information:**

The online version of this article (10.1007/s00066-024-02234-6) contains supplementary material, which is available to authorized users.

## Introduction

With ever-increasing precision of radiation modalities, i.e., particle therapy, and improved imaging enabling adaptive radiotherapy, profound understanding of the gross tumor volume (GTV) and the clinical target volume (CTV), which includes the GTV and microscopic tumor extension (MTE), prior to and during radiotherapy (RT) is crucial. This holds true for many solid tumors including esophageal cancer (EC), in which large CTVs have historically been used. These large volumes are based on previous histological assessments of the MTE in EC and calculations of the CTV margins required to encompass 95% of the MTE, which ranges from 6 to 24 mm around the GTV in EC [1–4]. However, these studies neither investigated the possible influence of the tumor microenvironment (TME) on MTE, nor the exact information on the in vivo localization of the specimens in the patients.

Factors of the TME, such as kinases, cancer stem cells, hypoxia, tumor cell proliferation, and immune cells, are hypothesized to be involved in MTE [5, 6]. FAK and ILK, known to be overexpressed in EC, are strongly linked to tumor cell survival, proliferation, invasion, and migration [7–9]. The cancer stem cell marker CD44 characterizes a tumor subpopulation highly associated with tumor metastasis and radioresistance in EC patients, ultimately leading to tumor recurrence [10, 11]. HIF-1α, an important biomarker of the hypoxic TME, contributes significantly to the survival of tumor cells and to resistance to radiochemotherapy (RCHT) [12, 13]. Lastly, Ki67 is a marker of cell proliferation and a well-known prognostic marker in a variety of solid cancers, including EC [14].

In a previous retrospective cohort of EC patients, we compared changes in the TME in patients with esophageal adenocarcinoma (AC) or squamous cell carcinoma (SCC) treated with neoadjuvant RCHT followed by resection (NRCHT+R) with those in patients who underwent resection only (R) [15]. However, these findings could not be directly correlated with MTE since the study was based on pre-existing paraffin-embedded tumor blocks, and their in vivo localization could not be reconstructed. For proton beam therapy of patients with EC, our department has introduced use of fiducial markers, which are endoscopically implanted at the cranial and caudal borders of the macroscopic tumor prior to RT planning [16]. Besides increased treatment precision, these fiducial markers, which remain in situ in the tumor resection specimen, hold information on the position of the former GTV in the paraffin-embedded tissue blocks and can be back-projected to the pretreatment computed tomography (CT) scans. Consequently, both the precise position of the tissue blocks and the potential postoperative tissue shrinkage impacting the MTE can be estimated.

Therefore, this study aimed at assessing various markers of the TME in a cohort of EC patients having undergone NRCHT+R and whose resection specimen contained implanted fiducial markers, and to correlate the possible MTE with the marker expressions.

## Materials and methods

### Study cohort and ethical considerations

This study included ten nonconsecutive patients with histologically confirmed EC (nine with SCC and one with AC) having undergone NRCHT+R at the University Hospital Carl Gustav Carus Dresden (UKD), Germany. The inclusion criteria for these analyses were patients who had (1) participated in the EVI study for fiducial marker placement (BO-EK-148042017) and undergone resection after NRCHT, (2) showed the presence of at least one fiducial marker on both the cranial and caudal GTV borders on postoperative imaging and in the resection specimen, and (3) had residual tumor after NRCHT+R. The Ethical Committee of the Dresden University of Technology (TUD), Germany, approved the study on 20.06.2022 (BO-EK-177042022). Written informed consent to use data for research purposes had been obtained previously from all patients.

### Patient characteristics and treatment regimen

After implantation of the fiducial markers during esophageal endoscopy [16], all patients underwent diagnostic [18F]-fluorodeoxyglucose positron-emission tomography and CT (FDG-PET-CT) within one to two weeks prior to NRCHT, which also served for radiation treatment planning purposes. Based on the information obtained from FDG-PET-CT and during endo-esophageal endoscopy, the GTV and CTV were defined following our internal guidelines: the CTV was created from the GTV using margins of 2.5 to 3 cm in the cranial and caudal directions, respectively, and a circumferential margin of 1.5 cm. Radiation treatment planning was performed using RayStation (versions 8B or 10B, RaySearch Laboratories AB, Stockholm, Sweden) applying an intensity-modulated proton therapy technique and correcting for the relative biological effectiveness (RBE) by a factor of 1.1. All ten patients received a total proton dose of 40 Gy (RBE) in 2‑Gy (RBE) fractions over the course of 4 weeks. Simultaneous chemotherapy was administered using carboplatin (AUC2) and paclitaxel (50 mg/m^2^ BSA) once weekly. All patients underwent surgery 5–7 weeks after the end of NRCHT. Tumor staging was performed according to the Union for International Cancer Control (AJCC/UICC, 8th edition) [17]. Treatment recommendations were made in the multidisciplinary tumor board of the NCT/UCC Dresden, and patients with cT3 and/or cN+ disease were treated with NRCHT+R; however, one of the patients with tumor stage cT2 cN0 underwent NRCHT due to suspicion of lymph node metastasis.

### Sample processing and image registration

Native resection specimens on ice were sent from the Department of Visceral, Thoracic, and Vascular Surgery to the Institute for Pathology, both of the UKD, immediately following tumor resection. The specimen was removed from fixation, opened, and pinned on corkboard with the endoluminal side facing upwards and collected for CT imaging (Somatom Definition AS, Siemens Healthineers, Erlangen, Germany). Navigational charts revealing the positions of fiducial markers within the specimen were produced from co-registered CT and photography using custom software implemented in Python/SciPy [18]. Tissue blocks were systematically cut and labeled on the chart by the pathologists, such that their exact in vivo position could be reconstructed. Afterwards, the blocks were placed into paraffin wax-embedding cassettes for further processing and storage.

### Immunofluorescence staining

Hematoxylin and eosin (H&E) staining of the resected tissue specimen after FFPE was conducted by the pathologists for histological assessment of the presence and location of viable tumor cells for diagnostic purposes (Supplementary 1).

**Fig. 1:**
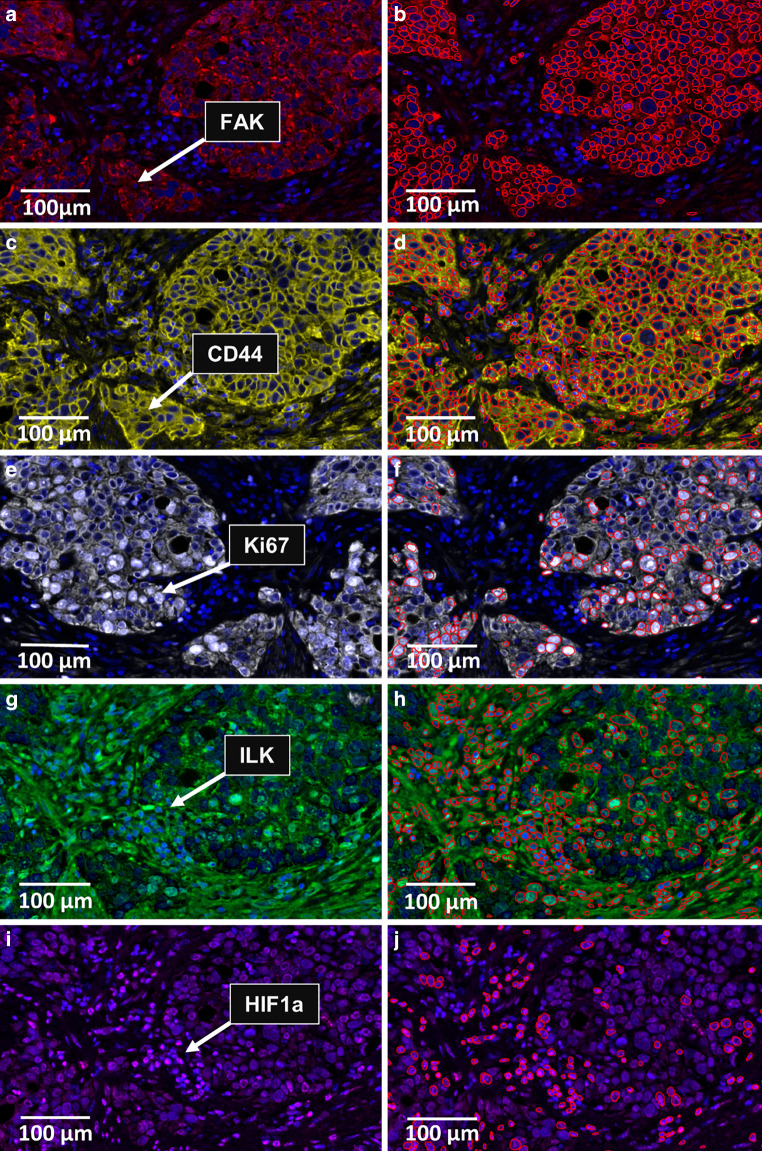
Multiplex immunofluorescence staining of tissue sections shows expression of tumor microenvironment markers. **a** Membrane expression (*red*) of FAK^+^ cells in tumor nests. **b** Membrane expression (*yellow*) of CD44^+^ cells in tumor nests and tumor stroma areas. **e** Nuclear expression (*white*) of Ki67^+^ cells in tumor nests. **g** Membrane expression (*green*) of ILK^+^ cells in tumor stroma areas. **i** Nuclear expression (*magenta*) of HIF-1α^+^ cells in tumor nests and tumor stroma areas. **b**,**d**,**f**,**h**,**j** *Red encircling* corresponds to the segmentation of marker positive cells

FFPE tumor blocks were subsequently obtained from the BioBank Dresden. On all FFPE blocks with residual tumor, multiplex immunofluorescence staining was established at the Institute of Immunology, Faculty of Medicine Carl Gustav Carus, TUD, Germany as previously described ([19]; Fig. 1; summarized in Supplementary 2).

### Image acquisition and analysis

Acquisition of the multiplex immunofluorescence staining images was performed using the Vectra 3 automated imaging system (Akoya Biosciences, Marlborough, MA, USA). Digital image analysis of the scans from all tumor sections was conducted in QuPath (version 0.3.2 University of Edinburg, UK) [20]. Since biomarker expression in the tumor stroma has repeatedly been shown to be a prognostic factor for overall survival and tumor progression in different cancer types [21], we hypothesized that higher expression of Ki67+, ILK+, HIF-1α +, CD44+, and FAK+ in tumor nests than in tumor stroma is associated with the depth of tumor infiltration.

Pan-cytokeratin (PanCK) expression in tumor tissue sections is a well-established diagnostic and prognostic marker in many solid tumors, including esophageal cancer [22, 23]. It has been utilized as a standard to detect and discriminate the intraepithelial PanCK^+^ tumor area from the stromal area in immunohistochemically stained resection tissues, thus enhancing TME assessment [24–26]. Therefore, in the first part of the image analysis, a deep learning algorithm based on PanCK staining was used to discriminate tumor nests (PanCK+) from tumor stroma (PanCK-) and to generate the corresponding annotations (tumor nest, tumor stroma), while removing areas of artifacts and autofluorescence. After tissue annotation, all cells were segmented in the DAPI nuclear-stained channel using the StarDist cell segmentation tool [27]. Next, classifier algorithms were trained for each individual marker, from which a composite classifier was generated. Quantification was based on the number of nuclear (Ki67^+^, ILK^+^, HIF-1α^+^) or membrane (CD44^+^, FAK^+^) stainings specific for each marker.

Before implementing each of the algorithms, a validation set of images was used to verify the reliability of the algorithm and results were checked for plausibility by two independent observers (BI, ET; Supplementary Fig. 2).

**Fig. 2:**
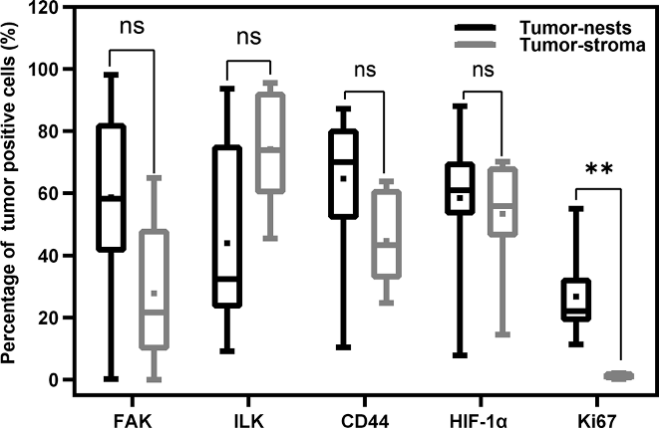
Boxplots showing the percentage of tumor cells positive for five markers of the tumor microenvironment within tumor nests and tumor stroma. Mann–Whitney test: ** *p* < 0.001, ns = not significant

The number of cells positively stained for each marker, including co-expression ratios, were extracted from QuPath using a script and percentages were further calculated in Microsoft-Excel (version 2016, Microsoft cooperation 2018, Redmond, WA, SA; see supplementary information for the script). This was done by dividing the total number of cells positive for each marker within a specific annotation (tumor nests or tumor stroma) by the total number of DAPI-stained cells with the same annotation.

### Calculation of the microscopic tumor extension

Patients’ pretreatment PET-CT images were exported as DICOM files and uploaded in the open-source software, 3D-Slicer (version 5.0.3 for Windows) [28]. The esophagus and fiducial markers implanted at the cranial and caudal tumor edges were segmented in 3D using the “fill between function” in the segment editor. Thereafter, the longitudinal distance between the fiducial markers representing the pretreatment in vivo macroscopic tumor was measured. Similarly, on the CT images of the tumor resection specimens revealing the ex vivo positions of fiducial markers, the distances between the fiducial markers were calculated. Finally, the tissue shrinkage rate (%) was calculated by dividing the ex vivo by the in vivo distances.

In patients with remaining tumor cells beyond the fiducial markers, thus resembling residual tumor in the CTV, the distance of the MTE was measured. For this, the tumor extension beyond the fiducial marker was established on the ex vivo FFPE tumor blocks (in millimeters). In order to translate this finding into the in vivo pretreatment situation, individual specimens’ shrinkage rates were multiplied by the calculated MTE and subsequently projected onto the PET-CT images using 3D-Slicer [28, 47].

### Statistical analyses

Statistical analyses were performed on the percentages of cells positive for the individual markers within the tumor nests and tumor stroma annotations from the specimens of each patient. All the graphs and statistical analyses were performed using GraphPad Prism software version 9.0 for Windows (GraphPad Software, San Diego, CA, USA). Mann–Whitney test was used to compare the expression of the markers between groups, and a *p*-value < 0.05 was considered significant. Bonferroni correction was used for multiple test adjustment. In addition, a heat map was created to show the co-expression between the markers.

## Results

The clinicopathological characteristics of the patients are outlined in Supplementary Table 1. Since there was only one patient with AC in this cohort, no comparative analyses of the markers of the TME in tumor nests or tumor stroma between the two tumor histologies were performed.

**Fig. 3:**
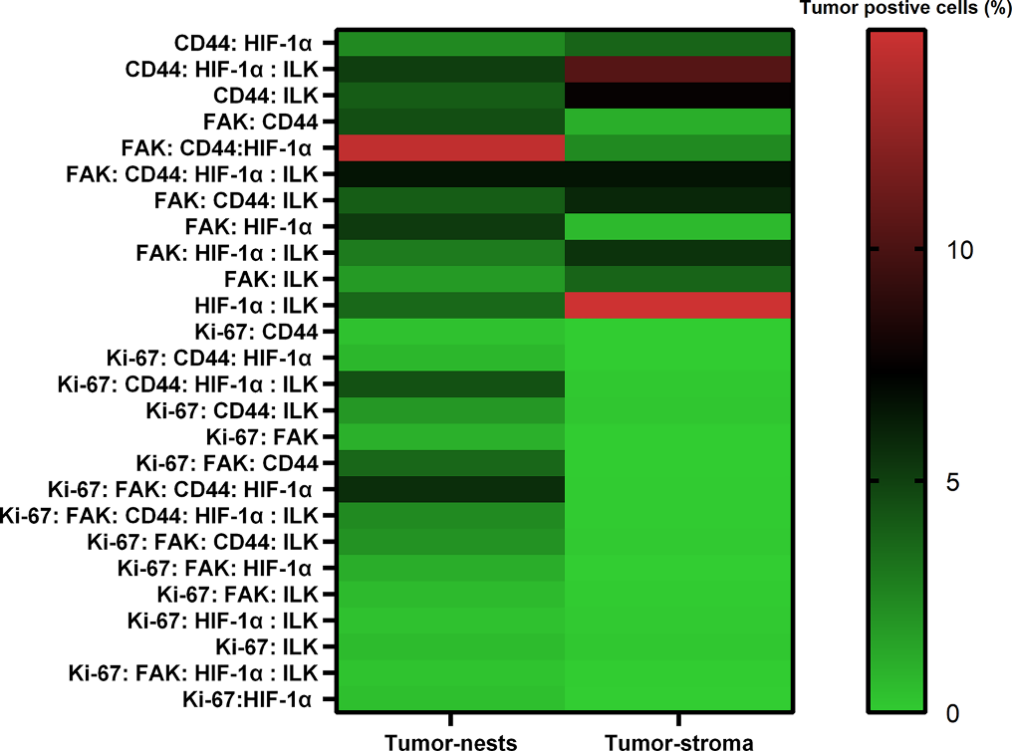
Co-expression of the five studied markers of the tumor microenvironment within tumor nests and tumor stroma of the ten included patients. The results are ordered with the highest expressions at the top and the lowest expressions at the bottom. The bar (*right*) depicts the percentage of tumor cells positive for a given marker

In tumor resection specimens of EC patients, the overall percentages of FAK^+^, CD44^+^, HIF-1α^+^, and Ki67^+^ cells were higher in tumor nests than in the tumor stroma, with Ki67^+^ cells reaching statistical significance (*p* < 0.001; Fig. 2). Conversely, expression of ILK^+^ cells was higher in tumor stroma compared to tumor nests, albeit not statistically significantly. In line with this, a heatmap illustrating the co-expression of markers of the TME showed more overall co-expression in tumor nests compared to tumor stroma (Fig. 3).

**Fig. 4:**
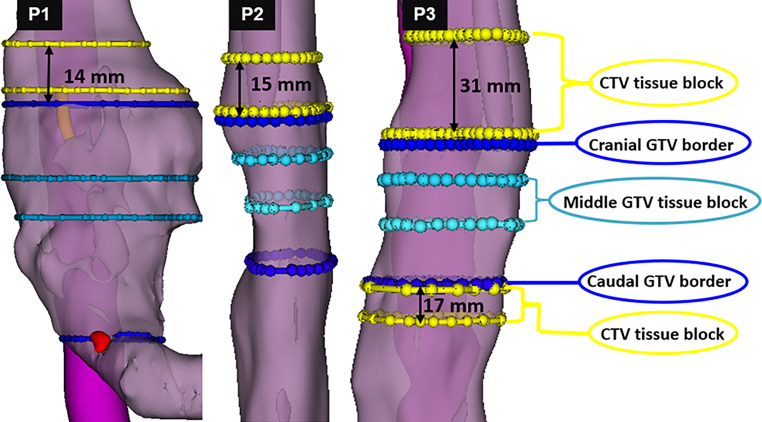
Patient-based microscopic tumor extension (MTE) beyond the gross tumor volume (*GTV*). P1 and P2 showed MTE of 14 and 15 mm in the cranial directions, respectively, in stage cT3 cN1 after NRCHT+R. P3 had MTE of 31 and 17 mm in the cranial and caudal directions, respectively, in stage cT3 after NRCHT+R

**Fig. 5:**
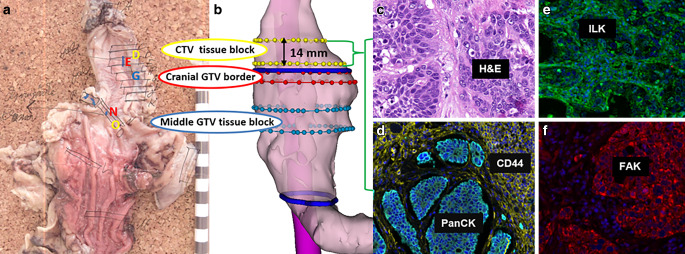
Patient-based image reconstruction of the microscopic tumor extension (MTE). **a** Resection specimen with demarcation of clinical target volume (*CTV*) block (*yellow*), gross tumor volume (*GTV*) border (*red*), and middle GTV block (*blue*). **b** Projection of the blocks’ origins onto the rendered esophagus taken from the treatment planning computed tomography. H&E staining (**c**) and immunofluorescence staining of tissue sections originating from the CTV block show cytoplasmic expression of PanCK in tumor nests and membrane expression of CD44 in tumor stroma (**d**), membrane expression of ILK in tumor nests (**e**), and membrane expression of FAK in tumor nests (**f**)

**Fig. 6:**
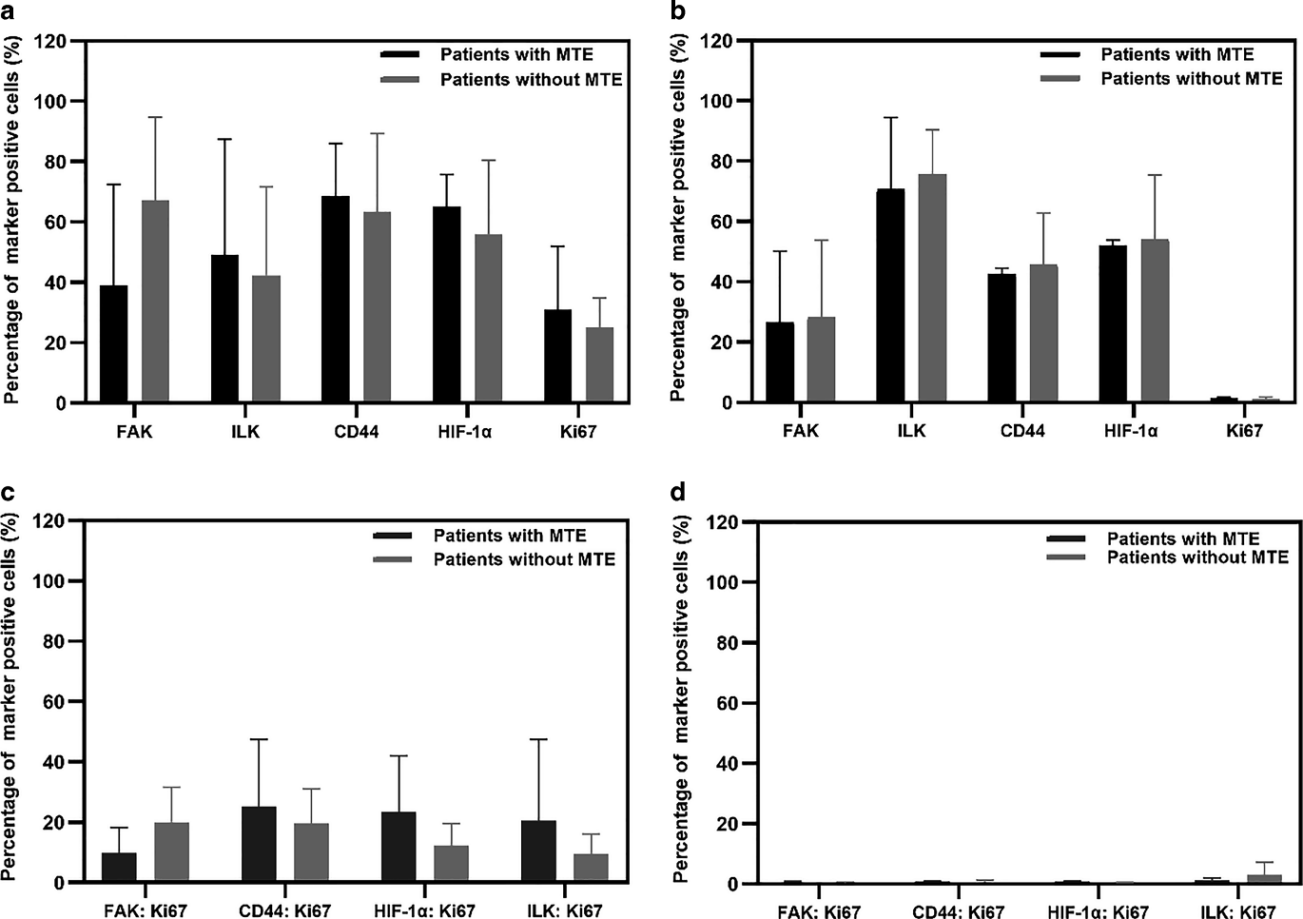
Comparison of expression of markers of the tumor microenvironment between patients with microscopic tumor extension (*MTE*) and those without MTE within **a** tumor nests and **b** tumor stroma. Moreover, the co-expression of TME markers within **c** tumor nests and **d** tumor stroma was investigated

Of interest, MTE beyond the fiducial markers was found in three patients (all cT3N1): two SCC patients (P1 and P2) showed MTE of 14 and 15 mm, and one AC patient (P3) still had MTE of 31 and 17 mm in the cranial and caudal directions, respectively (Fig. 4). In those patients, a stronger expression of FAK^+^, CD44^+^, and ILK^+^ cells was found in tumor nests between the fiducial markers (former GTV) and beyond (former CTV; Fig. 5), even after NRCHT. The expression of TME markers of these three patients in the former GTV versus the former CTV are shown for tumor nest and tumor stroma in Supplementary Fig. 3. Also, the expression of the assessed markers was compared between the three patients with MTE and the seven patients without MTE. However, no significant differences were found, neither for the tissue type (tumor nest, tumor stroma) nor upon combining the markers or using them separately (Fig. 6). Of note, a co-expression analysis of Ki67 with all the markers was done to determine the proliferation status of the selected TME markers even after NRCHT.

## Discussion

Overall, the results of this study showed higher expression of cells positive for the selected TME biomarkers (FAK^+^, CD44^+^, HIF-1α^+^, and Ki67^+^) within tumor nests compared to tumor stroma in tumor resection specimens of EC patients after NRCHT+R. Conversely, ILK-positive cells were highly expressed in the tumor stroma compared to the tumor nests. However, the marker expressions in the resection specimen of the three patients with residual MTE after treatment did not differ from those of patients without MTE, neither in the tumor stroma compared to the tumor nests, nor when combining marker expression.

To the best of our knowledge, this is the first study to thoroughly assess markers of the TME after NRCHT+R in EC patients and compare them to MTE. Some of our findings are in line with reports on the expression of markers of the TME in previous studies performed on pretreatment specimens in a variety of solid tumors, including EC, while others differ, as discussed below.

Previous studies using immunohistochemical analyses on tissues samples of patients with EC, colon cancer, breast cancer, and sarcomas compared the expression of FAK proteins in tumor compartments with normal tissues of either the same patients or between different patient cohorts [7, 29, 30]. These investigations showed higher expression of FAK in the cytoplasm of tumor cells and in the invasive front of tumor nests compared to the tumor stroma. Furthermore, higher expression of FAK was associated with tumor infiltration depth, lymph node metastases, and advanced disease stage, leading to poor prognosis and lower survival rates. Similar investigations comparing tumor and non-tumor tissue associated CD44 expression with tumor invasion, metastasis, progression, treatment resistance, and poor overall survival [31–33]. Many investigations on HIF-1α involvement in a hypoxic TME concentrated on its association with metastasis and biological response to treatment. Two studies using immunohistochemistry examined HIF-1α expression from resection tissues of ESCC patients who underwent RCHT [34, 35]. Their findings showed that high HIF-1α expression is significantly correlated with a poor degree of differentiation, lymph node metastases, and depth of invasion. Additionally, three studies assessed tissues samples of ESCC patients who underwent surgical resection using immunohistochemistry [36–38]. Thus, it was shown that high HIF-1α expression is associated with regional and distant tumor cell metastases, advanced tumor stage, depth of infiltration, positive surgical margins, and poor overall survival. Hu et al. [39] used immunohistochemistry to compare tumor tissues with adjacent normal tissues in patients with ESCC who underwent surgery. They revealed that HIF-1α expression was higher in cancer tissues than in surrounding normal tissues, and that higher HIF-1α expression was associated with tumor cell motility, recurrence, and poor prognosis. A few studies investigated the value of Ki-67 in predicting response in ESCC patients following NRCHT [14, 40–42]. These investigations found a link between a high Ki-67 index and a poorer postoperative survival rate. They indicated that elevated Ki67 expression in tumor tissues is a risk factor for tumor progression and poor survival in ESCC patients. Finally, contrary to our results, two immunohistochemical studies comparing tumor tissues with adjacent normal tissues of EC patients after resection demonstrated an overexpression of ILK in differentiated areas of tumor tissues as compared to adjacent normal tissues [9, 43].

Our findings revealed MTE of up to 31 and 17 mm in the cranial and caudal directions from the fiducial markers (GTV borders) after completion of NRCHT+R. However, both the treatment regimen and analysis method of our cohort differ from previous studies that investigated MTE in resection specimens of EC patients in order to estimate CTV margins for RT. Gao et al. [4] used H&E staining to examine MTE in surgical specimens of SCC or AC patients with pT1-4 EC treated by primary resection. SCC had a mean MTE beyond the GTV of 10.5 ± 13.5 mm and 10.6 ± 8.1 mm in the cranial and caudal directions, respectively, whereas AC had cranial and caudal MTE of 10.3 ± 7.2 mm and 18.3 ± 16.3 mm, respectively. In a further analysis, they found a statistically significant correlation between the extent of MTE and the pathological tumor stage. Based on these findings, they suggested that a craniocaudal CTV margin of 30 mm for SCC, and 30 mm cranial and 50 mm caudal for AC is adequate to cover MTE within the esophagus in 94% of patients. In a similar study, Song et al. [44] used H&E staining to estimate CTV margins in patients with pT2‑3 SCC of the esophagus having undergone esophagectomy. They reported MTE of 30–40 mm in either direction. To obtain 95% coverage of MTE, they advised CTV margins of 30 and 40 mm in the cranial and caudal directions from the GTV. Furthermore, two investigations used H&E staining to examine MTE in resection specimens of EC patients with pT1‑4 treated with surgical resection and reported cranial and caudal distances of 34 ± 22 mm and 40 ± 34 mm, respectively [45, 46]. In these studies, the MTE was not linked with the CVT margins.

Our study has some limitations, which may influence its findings. First, the study was based on a small sample size, due to the limited number of EC patients amenable to proton beam therapy, and to the labor-intense acquisition and processing of the resection specimen. Second, in some patients, only one fiducial marker could be implanted prior to treatment (stenosing tumor), or one of the two implanted markers was lost during NRCHT. Thus, fiducial markers were absent on pre- or posttreatment imaging and/or in the resection specimen, and those patients had to be excluded. The third limitation is the absence of a complementary cohort of patients who underwent resection alone. This cohort of resection alone would be important, since the length of MTE may be underreported in the studied cohort having undergone NRCHT+R. Finally, implantation of the fiducial marker used here is burdensome, such that replacement by a liquid fiducial marker is envisioned.

## Conclusion

Our findings indicate that in EC patients having undergone NRCHT+R, the overall expression of FAK, HIF-1α, Ki67, and CD44 was higher in tumor nests, whereas ILK was higher in tumor stroma. Differences in the TME between patients with residual tumor cells in the original CTV compared to those without were not found. We have successfully established a panel of TME markers and their link to MTE. However, there is insufficient evidence that the TME influences the required CTV margin on an individual patient basis.

### Supplementary Information

**Supplementary 1** **H&E staining protocol. **H&E staining was carried out using the automated H&E staining machine, Tissue-Tek Prisma® (Sakura Finetek Germany GmbH, Staufen, Germany) in the routine laboratory of the Institute for Pathology at the University Hospital Carl Gustav Carus. The FFPE tumor tissues were sectioned into 3 μm thick sections using a microtome and mounted on a slide (StarFrost, Engelbrecht GmbH—Medizin und Labortechnik, Edermünde, Germany) over a water bath. The sections were then dried at 60 °C for 20 min. The FFPE tissues were washed twice in xylene for 2.5 min each, followed by deparaffinization in series of descending alcohol (absolute ethanol, 96% ethanol, and 70% ethanol) for 1 min each and then washed in water for 30 s. In the subsequent step, the tissue was stained two times with hematoxylin (hematoxylin: Polyscience, Inc. Warrington, PA, USA) for 2.5 min each and washed in water for 2.5 min. The samples were then stained in alkaline eosin (eosin: Sigma-Aldrich, St Louis, MO, USA) for 3 min with a short wash in water for 10 s. Slides were dehydrated for 10 s in 70% ethanol and for 1 min each in 96% ethanol and absolute ethanol. The samples underwent a final treatment in xylene for 4.5 min and finally coverslipped with Tissue-Tek Film® (Sakura Finetek Germany GmbH).

Supplementary 2 Immunofluorescence staining protocol

**Supplementary Fig. 1** Workflow showing all stages of sample preparation: (a) an overview of inserted fiducial gold markers at the tumors’ cranial and distal borders prior to radiochemotherapy; (b, c) CT and photographs of resected specimen revealing positions of fiducial gold markers; (d) paraffin blocks of samples based on locations of gold markers; (e) H&E staining revealing the inserted fiducial gold marker

**Supplementary Fig. 2** Workflow showing multiplex immunofluorescence staining of tissue samples and the digital image analysis pipeline

**Supplementary Fig. 3** Comparison of the expression of markers of the tumor microenvironment within the former GTV and the former CTV in those three EC patients with residual microscopic tumor extension following NRCHT+R (P1, P2 & P3). Mann–Whitney test: ns = not significant

**Supplementary Table 1** Patient and treatment characteristics (*n* = 10)

**Supplementary Table 2** List of used antibodies, reagents, and dilutions

## Abbreviations

AC: Adenocarcinoma
AUC: Area under curve
BSA: Body surface area
CD44: Cluster of differentiation 44, a cancer stem cell surface glycoprotein
CROSS: Chemoradiotherapy for esophageal cancer followed by surgery study
CT: Computed tomography
CTV: Clinical target volume
EC: Esophageal cancer
ESCC: Esophageal squamous cell carcinoma
FAK: Focal adhesion kinase
FDG: Fluorodeoxyglucose
FFPE: Formalin-fixed paraffin-embedded
GTV: Gross tumor volume
H&E: Hematoxylin and eosin
HIF-1α: Hypoxia-inducible factor 1-alpha
ILK: Integrin-linked kinase
Ki67: Tumor proliferation nuclear protein, encoded by the *MKI67* gene
MTE: Microscopic tumor extension
NRCHT+R: Neoadjuvant radiochemotherapy plus resection
PanCK: Pan-cytokeratin
PET: Positron-emission tomography
R: Resection
RT: Radiotherapy
SCC: Squamous cell carcinoma
TME: Tumor microenvironment
TSA: Tyramide signal amplification
UKD: University Hospital Carl Gustav Carus Dresden
