# Supplementary material for: Correlation of microscopic tumor extension with tumor microenvironment in esophageal cancer patients
Source: Strahlenther Onkol. 2024 May 10;200(7):595–604. doi: 10.1007/s00066-024-02234-6 (PMC11186916; doi:10.1007/s00066-024-02234-6)
Supplement: Supplementary file 1 — Supplementary 1 H&E staining protocol. H&E staining was carried out using the automated H&E staining machine, Tissue-Tek Prisma® (Sakura Finetek Germany GmbH, Staufen, Germany) in the routine laboratory of the Institute for Pathology at the University Hospital Carl Gustav Carus. The FFPE tumor tissues were sectioned into 3 μm thick sections using a microtome and mounted on a slide (StarFrost, Engelbrecht GmbH—Medizin und Labortechnik, Edermünde, Germany) over a water bath. The sections were then dried at 60 °C for 20 min. The FFPE tissues were washed twice in xylene for 2.5 min each, followed by deparaffinization in series of descending alcohol (absolute ethanol, 96% ethanol, and 70% ethanol) for 1 min each and then washed in water for 30 s. In the subsequent step, the tissue was stained two times with hematoxylin (hematoxylin: Polyscience, Inc. Warrington, PA, USA) for 2.5 min each and washed in water for 2.5 min. The samples were then stained in alkaline eosin (eosin: Sigma-Aldrich, St Louis, MO, USA) for 3 min with a short wash in water for 10 s. Slides were dehydrated for 10 s in 70% ethanol and for 1 min each in 96% ethanol and absolute ethanol. The samples underwent a final treatment in xylene for 4.5 min and finally coverslipped with Tissue-Tek Film® (Sakura Finetek Germany GmbH). [file 66_2024_2234_MOESM1_ESM.docx]

**Supplementary 1 H&E staining protocol**

H&E staining was carried out using the automated H&E staining machine, Tissue-Tek Prisma® (Sakura Finetek Germany GmbH, Staufen, Germany) in the routine laboratory of the Institute for Pathology at the University Hospital Carl Gustav Carus. The FFPE tumor tissues were sectioned into 3 μm-thick sections using a microtome and mounted on a slide (StarFrost, Engelbrecht GmbH – Medizin und Labortechnik, Edermünde, Germany) over a water bath. The sections were then dried at 60°C for 20 min. The FFPE tissues were washed twice in xylene for 2.5 min each, followed by deparaffinization in series of descending alcohol (absolute ethanol, 96% ethanol, and 70% ethanol) for 1 min each and then washed in water for 30 s. In the subsequent step, the tissue was stained two times with hematoxylin (Hematoxylin: Polyscience, Inc. Warrington, PA) for 2.5 min each and washed in water for 2.5 min. The samples were then stained in alkaline eosin (Eosin: Sigma-Aldrich, St Louis, MO) for 3 min with a short wash in water for 10 s. Slides were dehydrated for 10 seconds in 70% ethanol and for 1 minute each in 96% ethanol and absolute ethanol. The samples underwent a final treatment in xylene for 4.5 minutes and finally coverslipped with Tissue-Tek Film® (Sakura Finetek Germany GmbH).
