# Supplementary material for: Correlation of microscopic tumor extension with tumor microenvironment in esophageal cancer patients
Source: Strahlenther Onkol. 2024 May 10;200(7):595–604. doi: 10.1007/s00066-024-02234-6 (PMC11186916; doi:10.1007/s00066-024-02234-6)
Supplement: Supplementary file 2 — Supplementary 2 Immunofluorescence staining protocol [file 66_2024_2234_MOESM2_ESM.docx]

**Supplementary 2 Immunofluorescence staining protocol**

Tyramide-signal amplification (TSA)-based Opal multiplex immunohistochemistry which can detect up to six co-localized epitopes (markers) on a single tissue section and emerged as an advanced technique for investigating the TME [19,42]. Here, we established and used a six-marker staining protocol (plus DAPI) to detect FAK, ILK, CD44, HIF-1α and Ki67 expressing cells.

FFPE tumor blocks from patients with EC were obtained from the BioBank Dresden, of the UKD. The FFPE tumor tissues were sectioned at 3 µm thickness. For each patient, multiplex immunofluorescence staining and analyses of all the markers presented here was performed on all FFPE blocks that were confirmed with primary tumor from histological diagnosis on Hematoxylin (Polyscience, Inc. Warrington) and Eosin (Sigma-Aldrich) staining for 40 and 30 minutes respectively. The automated Ventana Instrument (Discovery Ultra-0598775000, Roche, Basel, Switzerland) was used for performing the staining. After deparaffinization, antigen retrieval was performed in the cell conditioning 1 solution (Ventana Medical Systems, Tucson, AZ). Incubation of the primary antibody followed for 32 min at 36°C. Then, the appropriate secondary antibody (OmniMap anti-mouse or anti-rabbit ready-to-use (Ventana Medical Systems)) was applied for 12 min. Finally, one of the TSA fluorophores (Akoya Biosciences) was added to the tissue slides for 8 min. The primary and secondary antibodies were stripped off by heating the slides at 100°C in the cell conditioning 2 solution (Ventana Medical Systems) for 24 min. All steps except deparaffinization and antigen retrieval were repeated for each marker using a different TSA fluorophore for each primary antibody. Finally, tissue slides were counterstained with DAPI (Sigma Aldrich) and coverslipped with fluoromount medium (Southern Biotech, Birmingham, AL). The antibodies and other reagents used for the staining are summarized (see Supplementary Table 2)**.**
