# Supplementary material for: Correlation of microscopic tumor extension with tumor microenvironment in esophageal cancer patients
Source: Strahlenther Onkol. 2024 May 10;200(7):595–604. doi: 10.1007/s00066-024-02234-6 (PMC11186916; doi:10.1007/s00066-024-02234-6)
Supplement: Supplementary file 3 — Supplementary Fig. 1 Workflow showing all stages of sample preparation: (a) an overview of inserted fiducial gold markers at the tumors’ cranial and distal borders prior to radiochemotherapy; (b, c) CT and photographs of resected specimen revealing positions of fiducial gold markers; (d) paraffin blocks of samples based on locations of gold markers; (e) H&E staining revealing the inserted fiducial gold marker [file 66_2024_2234_MOESM3_ESM.docx]

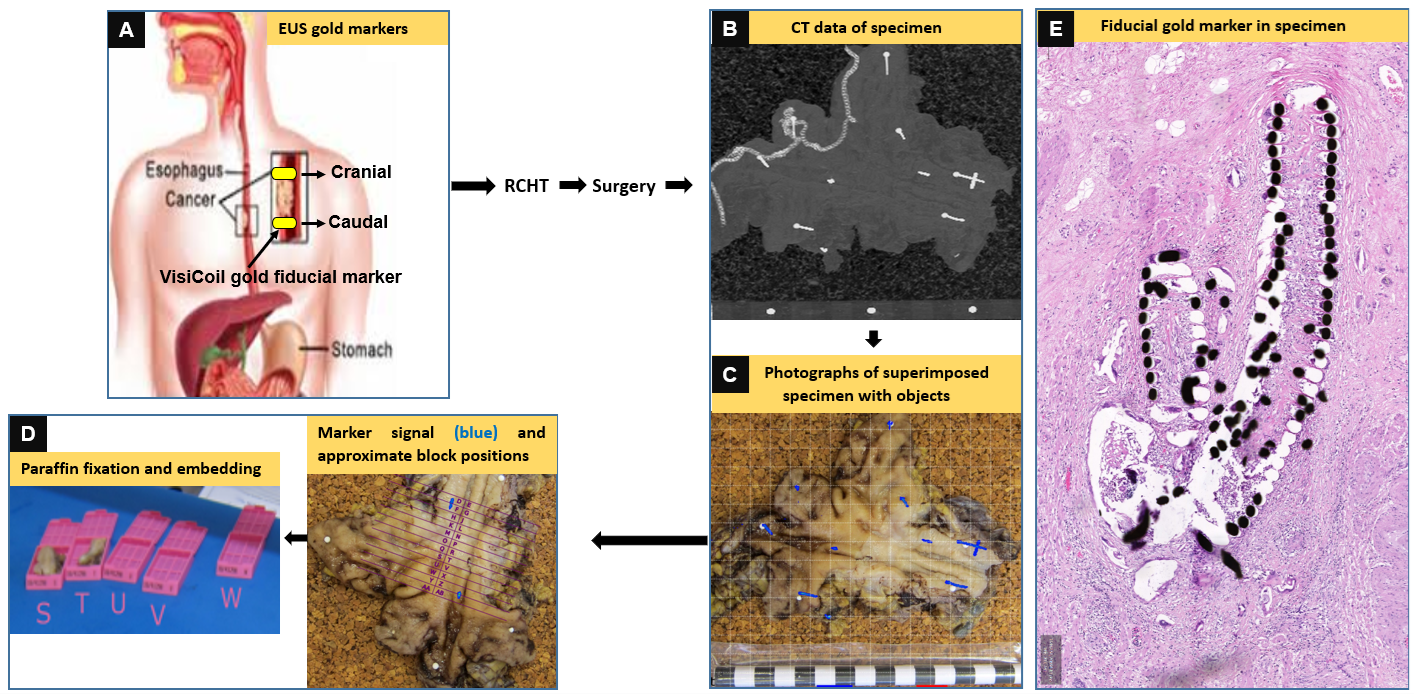


**Supplementary Figure 1** Workflow showing all stages of sample preparation: (a) an overview of inserted fiducial gold markers at the tumors’ cranial and distal borders prior to radiochemotherapy, (b, c) CT and photographs of resected specimen revealing positions of fiducial gold markers, (d) Paraffin blocks of samples based on locations of gold markers, and (e) H&E staining revealing the inserted fiducial gold marker
