# Supplementary material for: Correlation of microscopic tumor extension with tumor microenvironment in esophageal cancer patients
Source: Strahlenther Onkol. 2024 May 10;200(7):595–604. doi: 10.1007/s00066-024-02234-6 (PMC11186916; doi:10.1007/s00066-024-02234-6)
Supplement: Supplementary file 4 — Supplementary Fig. 2 Workflow showing multiplex immunofluorescence staining of tissue samples and the digital image analysis pipeline [file 66_2024_2234_MOESM4_ESM.docx]

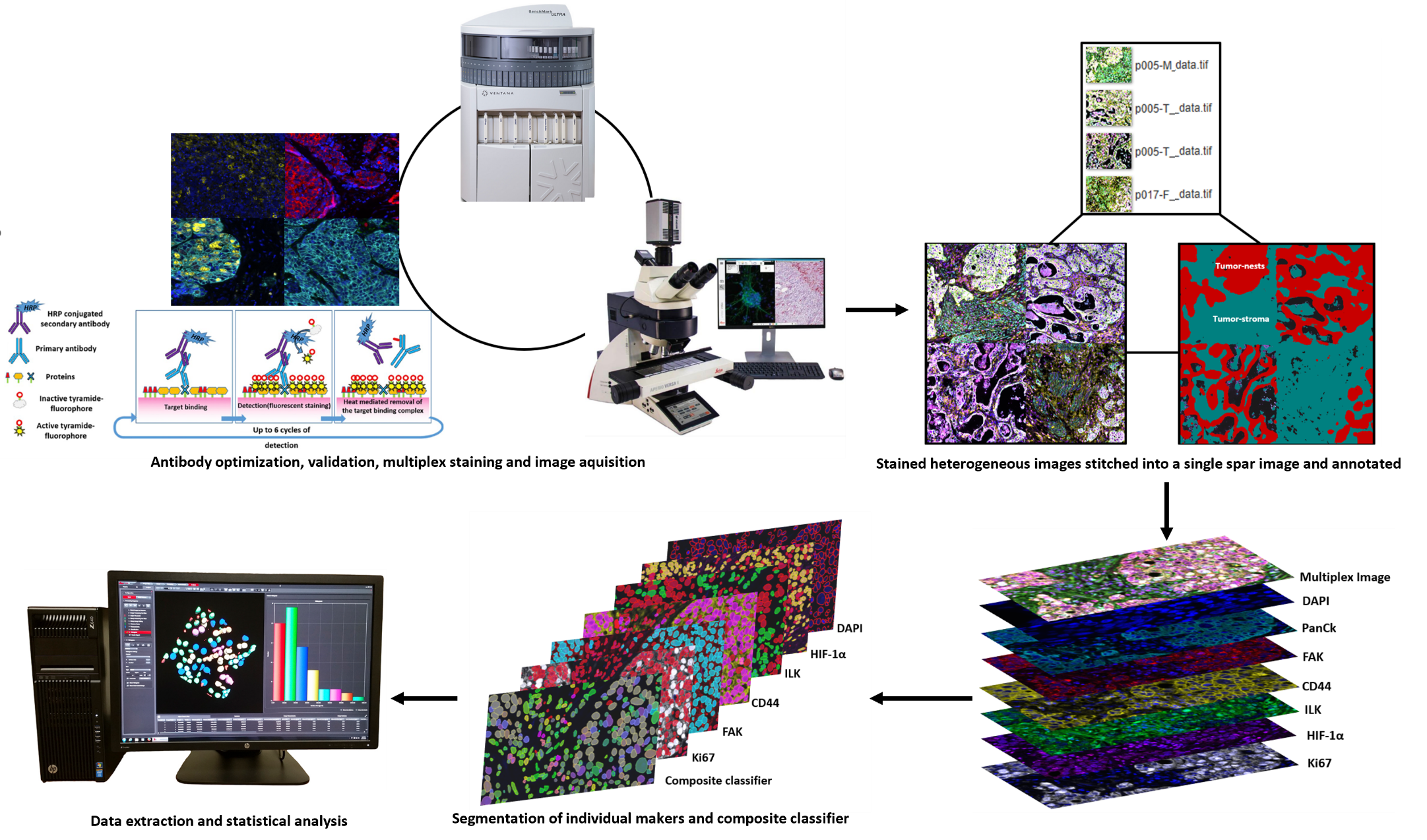
 **Supplementary Figure 2** Workflow showing multiplex immunofluorescence staining of tissue samples and digital image analysis pipeline
