# Supplementary material for: Correlation of microscopic tumor extension with tumor microenvironment in esophageal cancer patients
Source: Strahlenther Onkol. 2024 May 10;200(7):595–604. doi: 10.1007/s00066-024-02234-6 (PMC11186916; doi:10.1007/s00066-024-02234-6)
Supplement: Supplementary file 5 — Supplementary Fig. 3 Comparison of the expression of markers of the tumor microenvironment within the former GTV and the former CTV in those three EC patients with residual microscopic tumor extension following NRCHT+R (P1, P2 & P3). Mann–Whitney test: ns = not significant [file 66_2024_2234_MOESM5_ESM.docx]

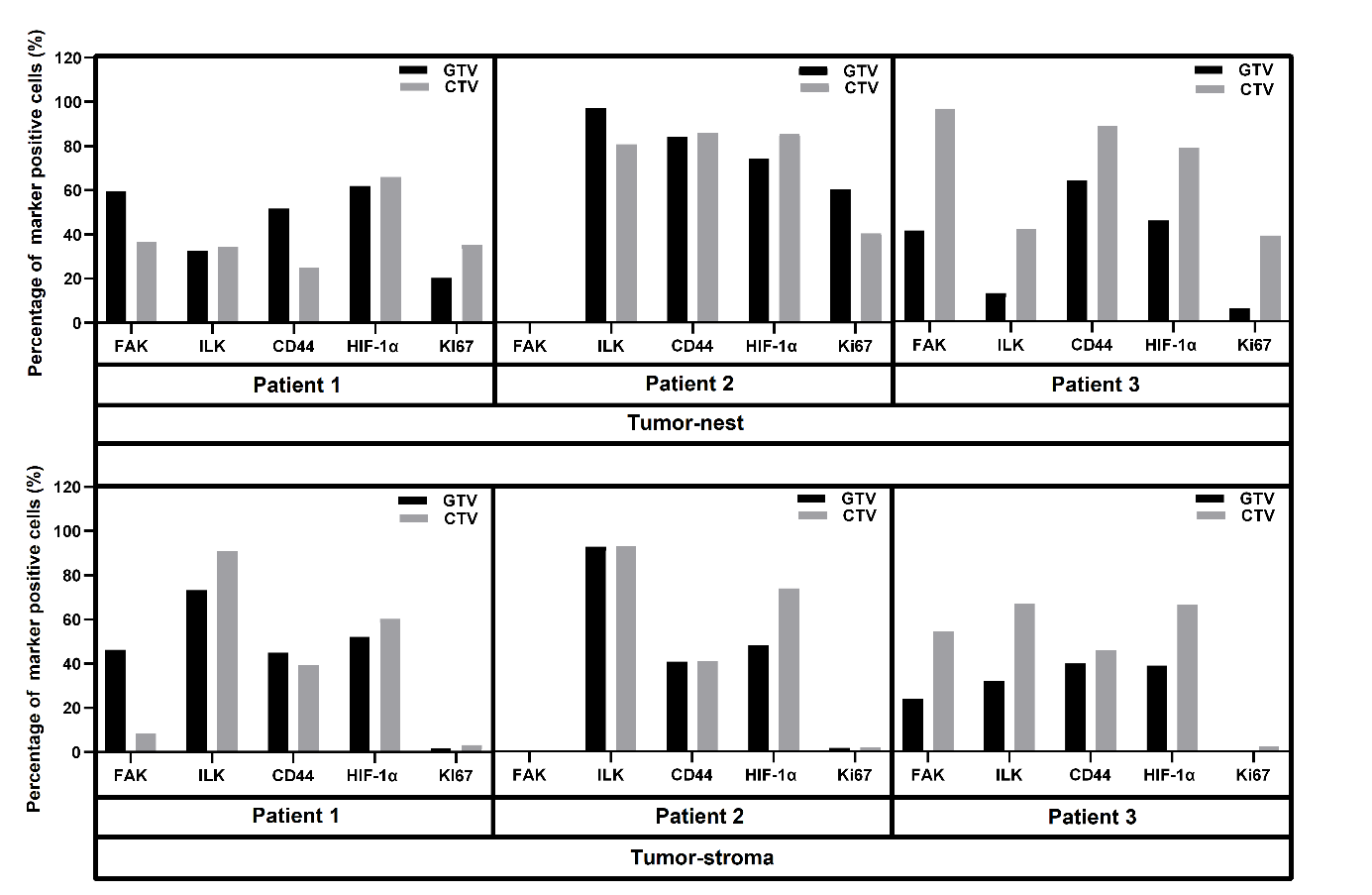
**Supplementary Figure 3** Comparison of the expression of markers of the tumor microenvironment within the former GTV and the former CTV in those three EC patients with residual microscopic tumor extension following NRCHT+R (P1, P2 & P3). Mann-Whitney test: ns=not significant
