# Supplementary material for: Correlation of microscopic tumor extension with tumor microenvironment in esophageal cancer patients
Source: Strahlenther Onkol. 2024 May 10;200(7):595–604. doi: 10.1007/s00066-024-02234-6 (PMC11186916; doi:10.1007/s00066-024-02234-6)
Supplement: Supplementary file 6 — Supplementary Table 1 Patient and treatment characteristics (n = 10) [file 66_2024_2234_MOESM6_ESM.docx]

| **Supplementary Table 1** Patient and treatment characteristics (**n=10**) | | | | | | | |
| --- | --- | --- | --- | --- | --- | --- | --- |
| **Patient number** | **Tumor**  **Type** | **Treatment** | **Gender** | **Age** | **TNM stage**  **(cT/cN)** | **RTx dose**  **Proton (Gy)** | **CTx agent** |
| 1 | SCC | NRCHT+R | M | 57 | cT3 cN1 | 2->40 | carboplatin; paclitaxel |
| 2 | SCC | NRCHT+R | M | 55 | cT3 cN1 | 2->40 | carboplatin; paclitaxel |
| 3 | SCC | NRCHT+R | M | 58 | cT2 cN1 | 2->40 | carboplatin; paclitaxel |
| 4 | SCC | NRCHT+R | M | 71 | cT3 cN1 | 2->40 | carboplatin; paclitaxel |
| 5 | AC | NRCHT+R | M | 61 | cT3 cN1 | 2->40 | carboplatin; paclitaxel |
| 6 | SCC | NRCHT+R | M | 58 | cT3 cN1 | 2->40 | carboplatin; paclitaxel |
| 7 | SCC | NRCHT+R | M | 54 | cT3 cN0 | 2->40 | carboplatin; paclitaxel |
| 8 | SCC | NRCHT+R | M | 52 | cT3 cN0 | 2->40 | carboplatin; paclitaxel |
| 9 | SCC | NRCHT+R | M | 59 | cT2 cN0 | 2->40 | carboplatin; paclitaxel |
| 10 | SCC | NRCHT+R | M | 52 | cT3 cN1 | 2->40 | carboplatin; paclitaxel |
|  |  |  | **Mean (Range)** | 57.7 (52-71) |  |  |  |
| **Abbreviations** AC=adenocarcinoma, SCC=squamous cell carcinoma, M=male, NRCHT+R=neoadjuvant radiochemotherapy followed by resection, cT=clinical tumor stage, cN=clinical lymph node stage. | | | | | | | |
