# Supplementary material for: Correlation of microscopic tumor extension with tumor microenvironment in esophageal cancer patients
Source: Strahlenther Onkol. 2024 May 10;200(7):595–604. doi: 10.1007/s00066-024-02234-6 (PMC11186916; doi:10.1007/s00066-024-02234-6)
Supplement: Supplementary file 7 — Supplementary Table 2 List of used antibodies, reagents, and dilutions [file 66_2024_2234_MOESM7_ESM.docx]

| **Supplementary table 2** List of used antibodies, reagents, and dilutions. | | | | |  |  |
| --- | --- | --- | --- | --- | --- | --- |
| **Primary Antibodies** | **Supplier** | **Cat. Number** | **Dilution** | **Secondary antibody** | **Fluorophore** | **Dilutions** |
| **Panel A** |  |  |  |  |  |  |
| CD44 (rabbit, MAb) | 5 | 5267145001 | 1:100 | OmniMap anti-Rb | 520 | 1:75 |
| FAK (mouse, MAb) | 3 | M723501-2 | 1:50 | HQ anti-Rb | 560 | 1:50 |
| HIF-1α (mouse, MAb) | 4 | NB100-105 | 1:50 | OmniMap anti-Ms | 570 | 1:75 |
| PanCK (mouse, MAb) | 2 | 37259S | pre | OmniMap anti-Ms | 540 | 1:150 |
| Ki67 (mouse, MAb) | 3 | M7240 | 1:100 | OmniMap anti-Ms | 650 | 1:150 |
| ILK1 (rabbit, MAb) | 2 | 3862S | 1:75 | OmniMap anti-Rb | 690 | 1:50 |
| **Secondary antibody** |  |  |  |  |  |  |
| OmniMap anti-Ms HRP | 5 | 5269652001 |  |  |  |  |
| OmniMap anti-Rb HRP | 5 | 5269679001 |  |  |  |  |
| HQ anti-Rb | 5 |  |  |  |  |  |
| **Other reagents** |  |  |  |  |  |  |
| Opal 520 Reagent Pack | 1 | FP1487001KT |  |  |  |  |
| Opal 540 Reagent Pack | 1 | FP1494001KT |  |  |  |  |
| Opal 570 Reagent Pack | 1 | FP1488001KT |  |  |  |  |
| Opal 620 Reagent Pack | 1 | FP1495001KT |  |  |  |  |
| Opal 650 Reagent Pack | 1 | FP1496001KT |  |  |  |  |
| Opal 690 Reagent Pack | 1 | FP1497001KT |  |  |  |  |
| Amplification Diluent | 1 | FP1498 |  |  |  |  |
| Antibody Diluent / Block, 1X | 1 | ARD1001EA |  |  |  |  |
| Fluoromount-G mounting medium | 7 | 0100-01 |  |  |  |  |
| Sigma DAPI | 6 | 32670-5mg |  |  |  |  |
| **Abbreviations** MAb=monoclonal antibody, Ms=mouse, Rb=rabbit, HRP=horseradish peroxidase. **Suppliers** 1. Akoya Biosciences, 2. Cell Signaling technologies, 3. DAKO 4. Novus Biological, 5. Roche Diagnostics, 6. Sigma-Aldrich, 7. Southern Biotech | | | | | | |
